# Respiratory Viral Infections in Athletes: Many Unanswered Questions

**DOI:** 10.1007/s40279-022-01660-9

**Published:** 2022-03-30

**Authors:** Olli Ruuskanen, Raakel Luoto, Maarit Valtonen, Olli J. Heinonen, Matti Waris

**Affiliations:** 1 Department of Paediatrics and Adolescent Medicine, Turku University Hospital and University of Turku, PL 52, 20521 Turku, Finland; 2 Research Institute for Olympic Sports, Jyvaskyla, Finland; 3 Paavo Nurmi Centre and Unit for Health and Physical Activity, University of Turku, Turku, Finland; 4 Institute of Biomedicine, University of Turku, Turku, Finland; 5 Department of Clinical Virology, Turku University Hospital, Turku, Finland

## Abstract

Upper respiratory tract infections (“common cold”) are the most common acute illnesses in elite athletes. Numerous studies on exercise immunology have proposed that intense exercise may increase susceptibility to respiratory infections. Virological data to support that view are sparse, and several fundamental questions remain. Immunity to respiratory viral infections is highly complex, and there is a lack of evidence that minor short- or long-term alterations in immunity in elite athletes have clinical implications. The degree to which athletes are infected by respiratory viruses is unclear. During major sport events, athletes are at an increased risk of symptomatic infections caused by the same viruses as those in the general population. The symptoms are usually mild and self-limiting. It is anecdotally known that athletes commonly exercise and compete while having a respiratory viral infection; there are no virological studies to suggest that such activity would affect either the illness or the performance. The risk of myocarditis exists. Which simple mitigation procedures are crucial for effective control of seasonal respiratory viral infections is not known.

**Supplementary Information:**

The online version contains supplementary material available at 10.1007/s40279-022-01660-9.

## Key Points

**Table:** 

Elite athletes are commonly considered prone to respiratory infections, but there are no high-quality long-term studies on the occurrence of etiologically defined viral respiratory tract infections in athletes, and the symptom prevalence, duration, and burden remain unclear.
We know too little of the factors affecting susceptibility to viral infections. Furthermore, the relative contribution of different transmission modes of different viruses is poorly understood. During the coronavirus disease 2019 pandemic, mitigation procedures in the population and in sports teams have proved effective. Which prevention strategies are crucial in athletes remains to be clarified.
This paper advocates conducting high-quality research in collaboration with infectious diseases and sports medicine communities to improve the knowledge on respiratory infections in athletes.

## Introduction

Elite athletes are generally believed to have an increased risk of respiratory viral infections [1–8]. However, there is no virological evidence supporting that view. Heavy exercise up, to 800 h yearly, is thought to weaken antiviral immunity [9–17]. In addition, an athlete’s immune system may be impaired by psychological stress, sleep disturbance, and nutritional restrictions [18–20]. The common occurrence of severe acute respiratory syndrome coronavirus 2 (SARS-CoV-2) in athletes has recently emphasized this issue [21–25].

The occurrence, clinical manifestations, health risks, and effects on performance of respiratory viral infections in elite athletes are poorly documented. No longitudinal studies with viral diagnostics have been performed, probably because of the financial cost, the logistical challenges, and the lack of collaboration between the researchers of infectious diseases and sports medicine. The enhanced susceptibility of athletes to respiratory viral infections has recently been the subject of debate [26, 27].

## Which Factors Affect Susceptibility to Respiratory Viral Infections?

**Fig. 1:**
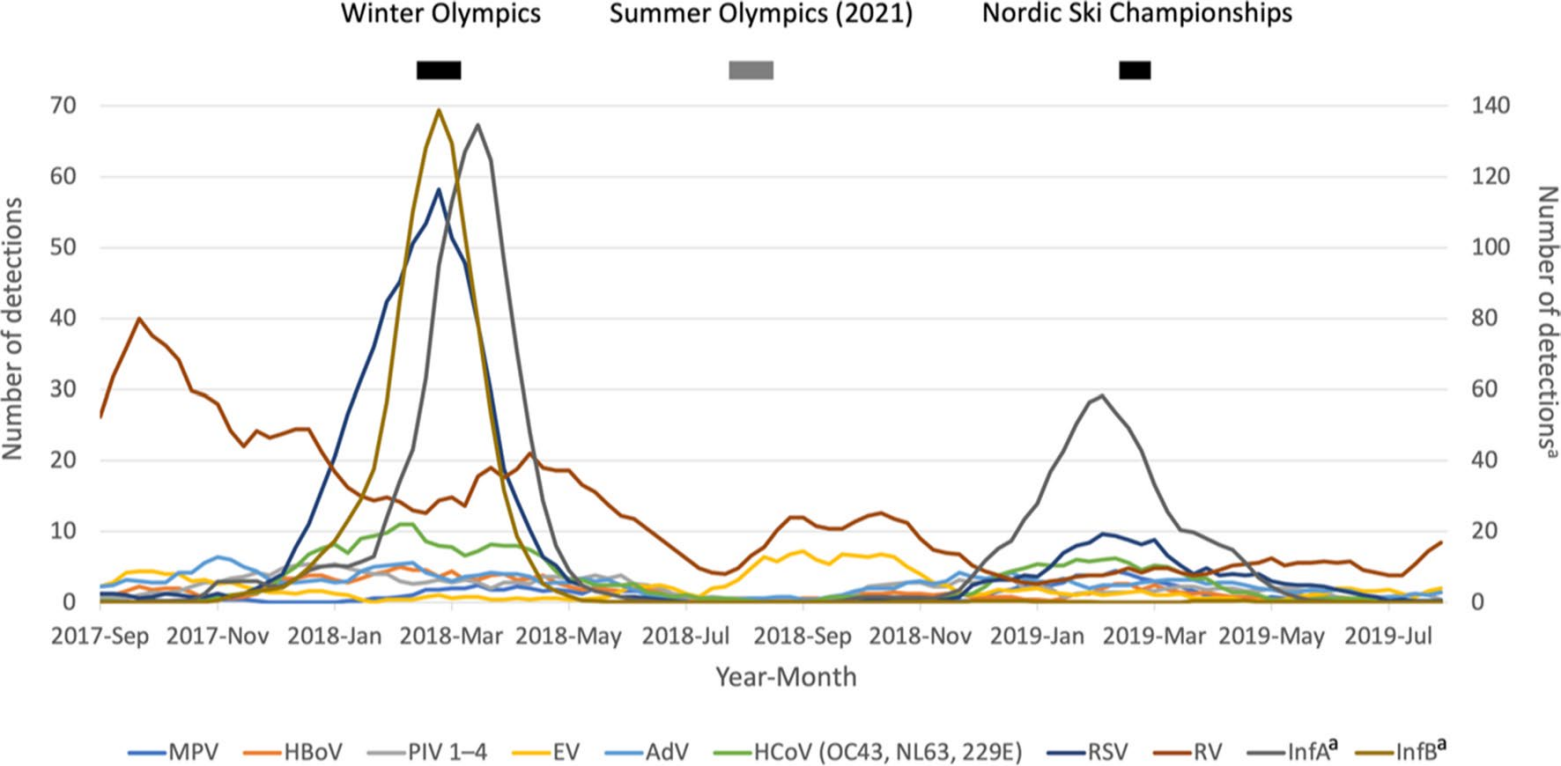
Seasonality of respiratory viruses in Turku, Finland, representing occurrence in the northern hemisphere. The figure shows the yearly viral highs and lows demonstrating the marked difference of the exogenous infection pressure in the community between winter and summer games. Many viruses are circulating at the same time. Virus detections are presented as the weekly 5-week running average from September 2017 (2017-Sep) to August 2019. Vertical bars represent the times of the Winter Olympics, Summer Olympics, and Nordic World Ski Championships. *AdV* adenovirus, *EV* enterovirus, *HBoV* human bocavirus, *HCoV* human coronavirus, *MPV* metapneumovirus, *Inf* influenza virus, *PIV* parainfluenza virus, *RSV* respiratory syncytial virus, *RV* rhinovirus. ^a^Secondary axis

Athletes’ susceptibility to respiratory viral infections is a complex interplay of many heterogeneous factors. Ten different viral species with hundreds of subtypes can induce respiratory infections. The clinical features of respiratory viral infections vary from one virus to another but overlap (Table 1). The etiology of an acute respiratory illness cannot be determined simply by clinical presentation. The risk of a respiratory infection is crucially dependent on the season, being highest during winter viral epidemics (Fig. 1). Of note, the transmission mechanisms of respiratory viral infections are not fully understood [28, 29].

**Table 1:** Viruses causing respiratory infections and their clinical presentations in young adults

	Common cold	Pharyngitis/tonsillitis	Bronchitis	Croup	Pneumonia
RNA viruses					
Rhinovirus, species A, B, C	++++	++	+	+	++
Coronavirus					
NL63, OC43, HKU1, 229E	+++	+	+	+	+
SARS-CoV-2	+++	+	+		++
Influenza virus types A, B, C	++	++	++	++	+++
Parainfluenza virus types 1–4	++	++	++	++++	+
Respiratory syncytial virus A, B	++	+	+	+	++
Human metapneumovirus	+	+	+	+	+
Enterovirus	+	+	+	+	+
DNA viruses					
Adenovirus	+	++	+	+	+
Human bocavirus	+	+	+	+	+
Epstein–Barr virus		+++			

In the order of frequency of causing the common cold in adults

Several nonimmunological factors affect an athlete’s risk of respiratory viral infections. They include living at home with young children, use of public transportation and international travel, human crowding, housing and socializing with other people, full-contact sports, and environmental factors, including temperature, humidity, ultraviolet radiation, and ventilation [21, 22, 27, 30–34].

## What is the Occurrence of Upper Respiratory Infections?

In the classic family studies conducted over 50 years ago, the annual frequency of acute upper respiratory infections in young adults was 2.3–4.8 [30]. In a recent study, 26 households with 105 individuals were followed for 1 year with weekly symptom diaries and nasal swabs for viral diagnostics. In 26 of the participants aged 18–39 years, the mean rate of respiratory illness episodes was 4.6, and a virus was detected in 6.3 episodes per person per year [35]. In an internet-based surveillance study, 125 participants aged 15–34 years reported an average of 3.7 acute respiratory illnesses during a 1-year period [36]. These data suggest that, in the general population, young adults experience two to six acute respiratory viral infections annually.

Most studies on the occurrence of respiratory infections in athletes have only included endurance sport disciplines and have been limited by variable illness criteria, self-reported illnesses, short follow-up periods, lack of control groups, and failing to include any studies of causative agents. Two long-term follow-up studies suggested that elite athletes were not significantly more prone to respiratory infections. During a 4-year study, 28 French elite swimmers experienced a yearly mean of 2.7 respiratory illnesses, as verified by a physician. The risk of illness was slightly increased with high-load training [37]. An analysis of self-reported infection events in 37 Norwegian cross-country skiers revealed a median of 3.0 respiratory tract events per year [38]. Of these athletes, 16% were illness prone, having six or more episodes per year. The winning athletes with the highest performance level reported fewer illness days than less successful athletes [38]. However, a review of 30 studies, including a total of 5471 runners, 2803 swimmers, and 1798 non-athletes, concluded that endurance athletes were more susceptible to respiratory infections than was a group of normally exercising controls [3]. Why some athletes develop frequent respiratory viral infections and others do not remains to be studied.

Increased incidences of symptoms of acute respiratory infection in athletes have been reported after a single long-lasting bout of exercise. In the well-known study on the 1987 Los Angeles Marathon, 12.9% of 2311 runners reported respiratory symptoms in the week after the race compared with 2.2% of 134 runners who did not participate in the race for reasons other than illness [39]. In the 2000 Stockholm Marathon study, 17% of 1694 runners reported an “infectious episode” during the 3 weeks before the race and 19% within 3 weeks after the race [40]. For the participants aged < 30 years, the incidence after the race was 25–27%. The marathon occurred in May, a time when the community pressure of respiratory viral infections in the Nordic countries is low (Fig. 1). Unlike the study investigators, we think that the young adult runners experienced an increased occurrence of respiratory illness. However, no control group was studied [40].

Upper respiratory tract infections are the most frequently reported illnesses during international athletic championships. During the London Summer Olympics, 7% of 10,568 athletes were reported to have an illness; most were suspected to be respiratory infections [41]. We find it implausible that studies of the 2010, 2014, and 2018 Olympic Winter Games, during the high season for respiratory viruses, only reported occurrences of respiratory illness in 2%, 4%, and 5% of the athletes, respectively [42–44]. These low prevalences are not in agreement with other observations. Of 44 Norwegian cross-country skiers, 48% became ill during or soon after the 10-day Tour de Ski [14]. During the 2018 Winter Olympic Games, 45% of 44 athletes in Team Finland experienced acute respiratory symptoms, as verified by the team physician [45]. During the 2019 Nordic World Ski Championships, 38% of 26 Finnish skiers experienced upper respiratory infections [46]. It seems obvious that sports teams are hesitant to report mild respiratory symptoms in team athletes.

## What is Known of Etiologically Defined Respiratory Viral Infections in Athletes?

As early as 25 years ago, we had already established the viral etiology in 69% of 200 young adults with a common cold. Rhinoviruses (52%) and coronaviruses (9%) were the most common etiologic agents [47]. Since then, new respiratory viruses (metapneumovirus, rhinovirus C, human bocavirus, and coronaviruses NL63, HKU1, and SARS-2) have been discovered. Sensitive and specific molecular test platforms for 16‒18 viruses, as well as molecular point-of-care tests have been widely available for many years [48]. With that in mind, it is strange that only three earlier studies on respiratory infections in athletes have investigated the viral etiology of the illnesses [49–51]. In two Australian studies, including 133 athletes with different summer sport disciplines, a viral etiology was detected in only 26% of the 98 infectious episodes (Table 2) [50, 51]. The low detection rates led to the commonly cited conclusion that noninfectious airway inflammation would cause the majority of respiratory symptoms in athletes. Using four different multiplex polymerase chain reaction panels, we determined a viral etiology in 23 (77%) of 30 winter sport athletes with acute respiratory symptoms [45, 46]. This recovery rate corresponds with many etiological studies carried out in the general adult population [47, 48].

**Table 2:** Studies reporting the occurrence of defined respiratory viral infections in athletes

Study	Athletes	Sport discipline	Study duration	Season; country	Respiratory infections	Viral etiology detected	Respiratory viruses^a^
Gundlapalli et al. [49]	46 symptomatic	Winter sport	2 weeks	Winter; USA	46	13 (36)	Influenza A or B 13
Spence et al. [50]	63	Triathlon, biking	5 months	Summer; Australia	28 (44)	7 (25)	Rhinovirus 4; adenovirus 2; parainfluenza virus 1; EBV 1
Cox et al. [51]	70 symptomatic	12 different	14 months	Year round; Australia	70	19 (27)	Rhinovirus 7; influenza viruses 7; parainfluenza viruses 4; coronaviruses 2; metapneumovirus 1; EBV 1
Valtonen et al. [45]	44	Winter sport	4 weeks	Winter; South Korea	20 (45)	15 (75)	Coronaviruses 5; RSV 5; metapneumovirus 4; influenza viruses 2; rhinovirus 1
Valtonen et al. [46]	26	Winter sport	2 weeks	Winter; Austria	10 (38)	8 (80)	Coronaviruses 4; rhinovirus 3; RSV 3

Data are presented as n or n (%) unless otherwise indicated

*EBV* Epstein–Barr virus, *RSV* respiratory syncytial virus

^a^Some athletes had dual viral infections

In the five etiological studies conducted in athletes, the most frequently detected viruses were rhinoviruses, seasonal coronaviruses, influenza viruses, and respiratory syncytial viruses, similar to in the general population with acute respiratory illnesses (Table 2) [45, 46, 49–51]. Interestingly, athletes do not seem to experience adenovirus infections. In contrast, military trainees most commonly experience adenovirus infections. They are the same age and share the same risk factors, such as heavy physical and mental stress and shared housing [52]. These observations do not support the view that respiratory viral infections in athletes would be opportunistic infections (infections that occur more often in people with a weakened immune system than in people with a healthy immune system) as stated in some exercise immunology studies. A recent study in Finnish elite cross-country skiers found no increased replication of persistent viruses typically activated in immunosuppressed individuals [9].

To test or not to test for viruses is a well-justified question when treating athletes with respiratory infections. Traditionally, the main value of viral testing has been to differentiate between viral and bacterial infections. Testing should reduce unnecessary antibiotic use, which has been too common in elite athletes. Antiviral therapy is only available for influenza. Prompt viral diagnosis permits isolation and cohorting of athletes, thereby mitigating the risk of transmission. Detection of the causative virus enables gathering of information about the major transmission mechanisms, incubation periods, generation times, clinical profiles, and duration of infectiousness of the particular virus. During our experience with virus diagnostics in elite athletes, we have learned that naming the causative virus markedly increases an athlete’s satisfaction. However, it must be emphasized that testing symptomatic cases on site and isolating them is not sufficient to control the transmission of respiratory viral infections in a sports team (Fig. 1 in the electronic supplementary material [ESM]) [45, 46].

## What are the Transmission Risk Factors?

Many behavioral factors may enhance the risk of respiratory viral infections in athletes more than traditionally suggested exercise-associated immunological disturbances [26]. Respiratory virus infections are mostly spread by means of close physical contact through the air and on surfaces [28]. Athletes are more exposed than the general population because of increased verbal interaction and close physical contact during travel, shared housing, indoor spaces with poor ventilation, meal sharing in restaurants, high-contact-risk sports, and mass gatherings.

There is also a risk of respiratory illness transmission during commercial air travel. Respiratory viruses have been detected in different places at airports [53]. In-flight transmission of influenza virus and SARS-CoV-2 is well established [54, 55]. Athletes traveling over more than five time zones have a two to threefold increased risk of illness [56]. In Norwegian cross-country skiers, the single greatest risk factor for infections was international air travel [38]. In our studies, ten of 182 sport team members were shown to be ill with different respiratory viral infections while traveling to international games [45, 46]. Some infections spread during the flight to neighboring team members [45]. The accepted rule of a safe distance of more than two seat rows may not be valid [57].

During Olympic Games, athletes share housing with four to six other athletes and common lounges, which mimics living in a household where respiratory viral infections are transmitted effectively. The secondary attack rate in households is usually 20–40% depending on the virus [58, 59]. Shared housing is not generally understood as a risk factor for athletes (Fig. 2 in the ESM). However, during the 2018 Winter Olympics, the successful Norwegian team stayed in a hotel outside the Olympic village, and the athletes occupied single or double rooms.

A competition situation seems to predispose athletes to respiratory viral infections. Competitions were a major risk factor in the study of Norwegian cross-country skiers [38]. In a recent study, winter sport athletes had a sevenfold increase in the risk of respiratory illness during a 2-week international championship when compared with control subjects living and exercising normally. The risk was twofold when compared with the support staff, who shared many risk factors such as traveling, shared housing, and crowding [46].

## What are the Clinical Manifestations of Respiratory Viral Infections in Athletes?

The relative immunosuppression of athletes would be expected to be associated with more pronounced illness. However, symptoms are usually mild and the same as those recorded in the general population: sore throat, sneezing, rhinitis, nasal congestion, and cough. Fever is uncommon [45, 46]. Among elite skiers, 20% of the viral infections were asymptomatic [46]. The average duration of symptoms is 5–9 days [38, 45, 46, 50, 51]. In 199 young adults from the general population, the mean duration of a common cold was 10 days [60]. The viral loads in athletes are mostly low and viral shedding is short, which does not support significant suppression of immunity. Significantly, athletes seem to seldom experience bacterial respiratory infections (acute otitis media, tonsillitis, sinusitis, pneumonia), and antibiotic treatment is rarely indicated [45, 46, 50, 51].

## What Have We Learned from COVID-19 in Athletes?

SARS-CoV-2 differs in many aspects from other respiratory viruses. SARS-CoV-2 primarily spreads in poorly ventilated indoor spaces through air exhaled when infected people sneeze, cough, breathe, talk, shout, or sing [61, 62]. The transmission is greater the closer a person is to the source of exhalation. COVID-19 outbreaks have been reported, for example, in soccer, ice hockey, and basketball teams. In a UK study of 147 athletes with COVID-19, the most prevalent symptoms were fatigue (57%), dry cough (50%), and headache (46%). The median duration of symptoms was 10 days [63]. In a multicentre investigation of the major North American professional sport leagues, 789 athletes with COVID-19 were studied. None of the athletes were clinically assessed as having severe COVID-19 illness; 58% had mostly mild symptoms, and 42% were paucisymptomatic or asymptomatic [23]. In a study of 137 collegiate athletes, 82% were symptomatic but experienced only mild (67%) to moderate (33%) symptoms, most frequently loss of smell or taste, fever, headache, and fatigue [64]. In the UK study, a quarter of 147 athletes had not returned to full sport participation at 28 days after symptom onset [63]. Persistent shedding of SARS-CoV-2 among 3648 individuals participating in the US National Basketball Association closed campus was detected in only 36 (1%) cases, most of them presumably elite basketball players [65]. The occurrence of long COVID among elite athletes is not known.

Myocarditis is the most concerning potential consequence of COVID-19 in athletes. In three studies, 789 professional, 1597 university, and 3018 collegiate athletes underwent cardiac triad testing (electrocardiography, cardiac troponin, echocardiography), followed by cardiac magnetic resonance testing when indicated as necessary by the screening tests. Cardiac involvements were identified in 5 (0.6%), 37 (2.3%), and 21 (0.7%) athletes, respectively [23–25]. These observations support the view that—like non-COVID respiratory viral infections—COVID-19 is a mild illness in elite athletes. The great majority of athletes will recover from SARS-CoV-2 infection uneventfully and may return to sport without cardiac testing. Cardiac triad testing is recommended for athletes with cardiopulmonary symptoms [23–25].

## Are There Risks to Health While Exercising with Respiratory Viral Infection?

It is logical to think that heavy physical stress during an ongoing viral infection would increase the duration and severity of illness and cause complications. However, in an experimental study, young adults were infected with rhinovirus 16 and then engaged in a 10-day moderate-intensity exercise program. Exercise did not increase the duration or severity of the infection compared with controls [66]. It is well known to sport physicians that athletes train and compete while experiencing respiratory viral infections. For example, in the National Hockey League, only fever sanctions an exemption from playing. Studies conducted during major winter events suggest that competing during a respiratory viral infection does not increase its duration or severity [45, 46]. On the other hand, acute infective illness may reduce performance [67].

A viral illness may provoke myocarditis. In the German registry of sports-related sudden cardiac arrest (*n* = 349) over a 6-year period, myocarditis was detected in 13 (3.7%) young adults. The myocarditis was preceded by upper respiratory tract infections in most of the affected patients [68]. During a 27-year period, of the 1049 sudden cardiac deaths of US athletes, 41 (3.9%) cases were caused by myocarditis [69].

Different return-to-sport protocols have been recommended according to expert opinion. Athletes are advised to rest for a period from 1 day up to 1 week after a febrile illness. According to “a neck-check rule”, exercise is allowed if symptoms are limited to the upper respiratory system [70]. This rule lacks supporting scientific evidence, may even be hazardous, and should be abandoned. Viruses that are potentially cardiogenic or affecting the central nervous system, such as enteroviruses, may induce only minor upper respiratory symptoms [71]. Decisions about the amount of time necessary to avoid sports practice should be based on common sense, the individual, the symptoms, and the response to a gradually increased exercise regimen.

## Can Respiratory Viral Infections be Prevented?

Prevention of respiratory viral infections in athletes is a complex issue because transmission is affected by the virus, the host, the behavioral and environmental characteristics, and—most importantly—the exogenous infection rate in the community (Fig. 1). Resistance is a combination of many personal (behavioral and immunity-enhancing) and shared responses (Table 3). There is no high-quality evidence of the efficacy of a single intervention or a combination of interventions for managing antiviral immunity or viral transmission in athletes.

**Table 3:** Strategies recommended to prevent respiratory viral infections in athletes

Strategies
*To minimize viral transmission*
In everyday life
Be aware of high-risk viral seasons Avoid individuals with common colds Use a fist bump instead of a handshake Remember careful hand hygiene
During traveling and competitions
Universal masking Minimize shared housing and meal sharing Avoid close physical contacts and crowds Avoid high-touch surfaces; use disinfection Isolate when you have a common cold
*To manage antiviral immunity*
Balance training load and recovery Avoid undernutrition and keep a relative energy balance Use evidence-based supplements (vitamins D and C) Regularly sleep 7–8 h per night Use professional guidance to maintain a proper diet Manipulate gut microbiome with prebiotics, probiotics, and postbiotics Get vaccinated (e.g., pneumococcal, influenza, COVID-19)

*COVID-19* coronavirus disease 2019 (caused by severe acute respiratory syndrome coronavirus 2 [SARS-CoV-2])

Major international sport competitions offer an ideal real-world setting in which to study the occurrence and transmission dynamics of respiratory viral infections. Studies showed that 30–40% of elite athletes' had symptomatic respiratory viral infections during a 2- to 3-week period of major games [14, 45, 46]. The 2021 Nordic World Ski Championships were organized during the COVID-19 pandemic. Strict COVID-19 mitigation strategies (e.g., universal masking, maintaining physical distance, enhanced hand hygiene) were carried out at the individual, team, and community levels [7, 72, 73]. During the 2 weeks of the games, no cases of symptomatic respiratory infection were identified by the team physicians in 76 members of Team Finland. This preliminary observation agrees with many studies showing that the mitigation strategies used during COVID-19 were also associated with a dramatic decrease in the occurrence of non-COVID respiratory viral infections [74]. Have we finally learned to control the common cold? The key questions remain, “What mitigation procedures are crucial for the effective control of respiratory viral infections, and, significantly, would they be socially acceptable?”.

## Conclusion and Unanswered Questions

We advocate for the conduct of high-quality research in collaboration with infectious diseases and sports medicine communities to improve the knowledge of respiratory infections in athletes. This research should be directed at answering the following questions:

- What is the annual incidence of respiratory viral infections in elite athletes and does it differ from that in normally exercising young adults?
- What are the precise mechanisms behind the exceptional susceptibility of athletes to respiratory viral infections during major international championships? Is this susceptibility immunological, psychological, or behavioral or a combination of all three? Are the infections acquired in the homeland or during travel and via the on-site community or the team?
- What are the clinical manifestations of different respiratory viral infections in elite athletes?
- What is the clinical significance of asymptomatic respiratory viral infections in athletes?
- How much do different symptomatic respiratory viral infections affect athletes’ performance?
- Is exercising while ill a risk for an athlete’s health and performance?
- How should the decision of return to play after an acute respiratory viral infection be determined?
- What measures can effectively prevent respiratory viral infections and their spread in athletes?

## Supplementary Information

Below is the link to the electronic supplementary material.

Suppl. Fig. 1 Spread of respiratory viruses in Team Finland during the 2018 Winter Olympics. The figure demonstrates the gradual transmission of different respiratory viruses within the team, indicating introduction from outside the team in most occasions. Inf, influenza virus; RSV, respiratory syncytial virus; RV, rhinovirus; HCoV, human coronavirus; MPV, human metapneumovirus; HBoV, human bocavirus. (JPG 809 kb)

Suppl. Fig. 2 The room plan of shared housing of 6 athletes of Team Finland during the 2018 Winter Olympic Games. The figure demonstrates the risk of transmission of viral infections in the unit. The rectangles illustrate the location of the beds. The numbers indicate the sequence of the infections. Athlete 1 developed gastroenteritis (G-itis) on February 6. He was isolated. On February 11, athletes 2 and 3 moved to the apartment, both reported mild respiratory symptoms but were negative in the point-of-care test for respiratory syncytial virus (RSV) and influenza A virus. Two days later, athlete 3 developed fever and was positive for the influenza B virus (InfB). He was isolated. Oseltamivir prophylaxis was initiated for the other athletes. On February 16, athlete 5 reported nasal congestion and was positive for RSVb. He stayed isolated in his room and his roommate, athlete 6, moved. Five days later he developed nasal congestion and was positive for RSVb. Two athletes, stayed healthy (H). (JPG 743 kb)
