# Supplementary figures and images for: Respiratory Viral Infections in Athletes: Many Unanswered Questions
Source: Sports Med. 2022 Mar 30;52(9):2013–21. doi: 10.1007/s40279-022-01660-9 (PMC8965548; doi:10.1007/s40279-022-01660-9)

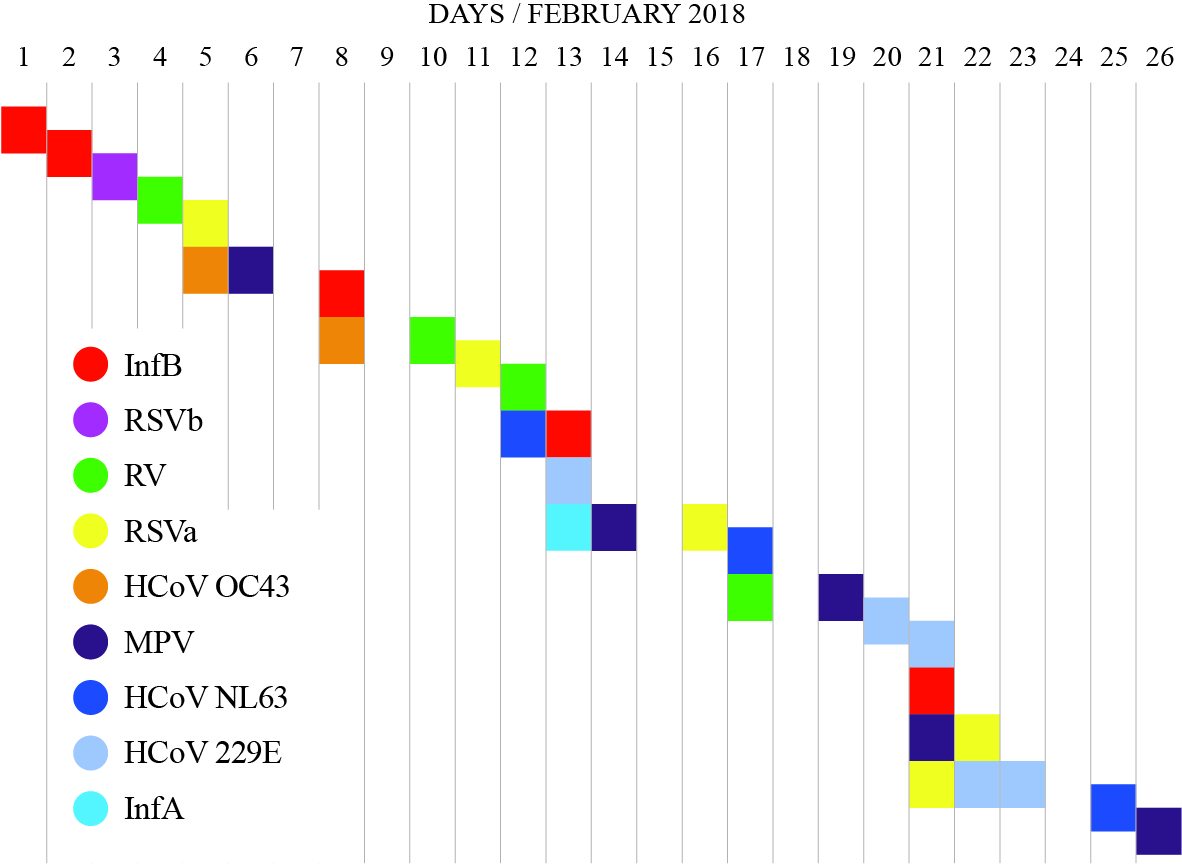

Supplement: Supplementary file 1 — Suppl. Fig. 1 Spread of respiratory viruses in Team Finland during the 2018 Winter Olympics. The figure demonstrates the gradual transmission of different respiratory viruses within the team, indicating introduction from outside the team in most occasions. Inf, influenza virus; RSV, respiratory syncytial virus; RV, rhinovirus; HCoV, human coronavirus; MPV, human metapneumovirus; HBoV, human bocavirus. (JPG 809 kb) [file 40279_2022_1660_MOESM1_ESM.jpg]

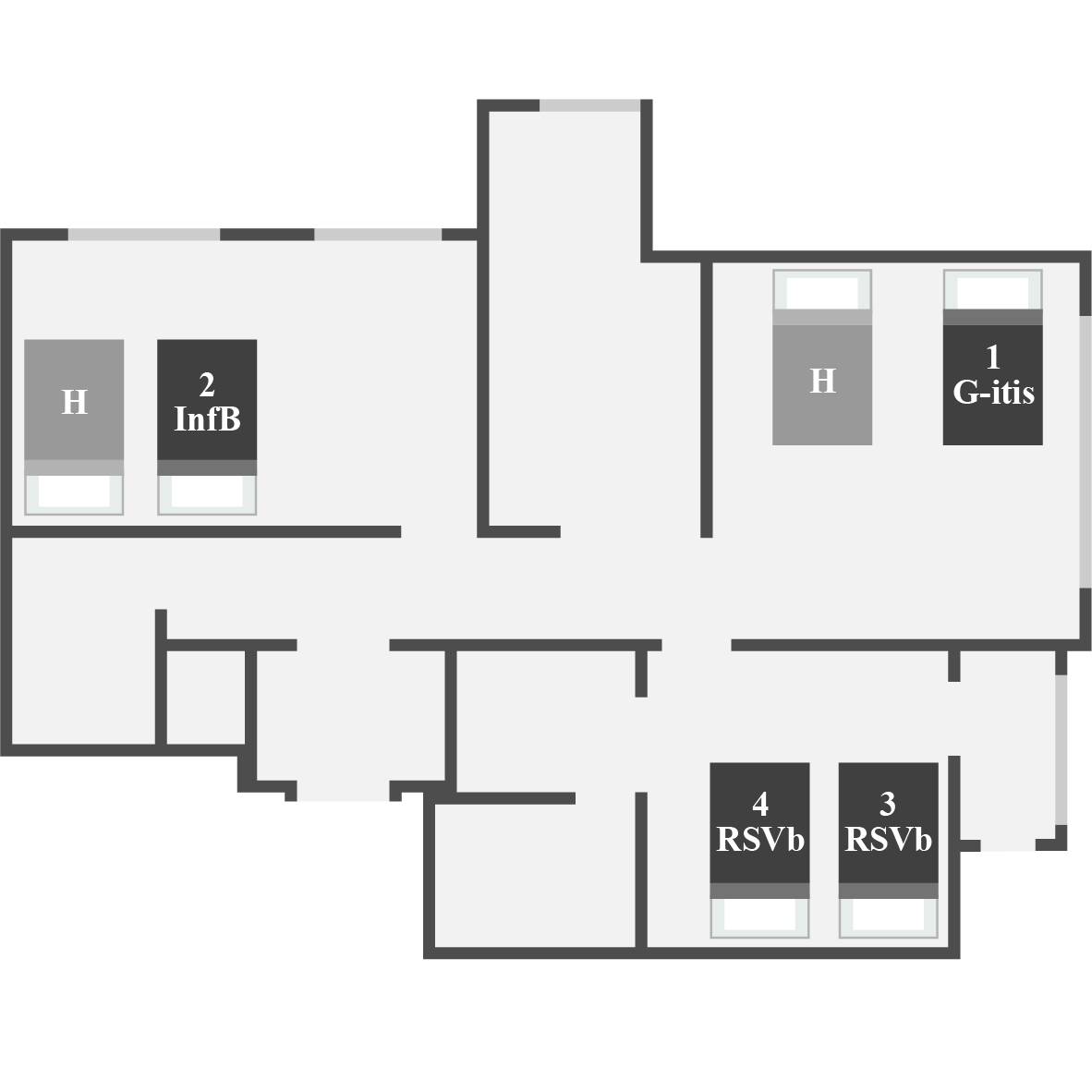

Supplement: Supplementary file 2 — Suppl. Fig. 2 The room plan of shared housing of 6 athletes of Team Finland during the 2018 Winter Olympic Games. The figure demonstrates the risk of transmission of viral infections in the unit. The rectangles illustrate the location of the beds. The numbers indicate the sequence of the infections. Athlete 1 developed gastroenteritis (G-itis) on February 6. He was isolated. On February 11, athletes 2 and 3 moved to the apartment, both reported mild respiratory symptoms but were negative in the point-of-care test for respiratory syncytial virus (RSV) and influenza A virus. Two days later, athlete 3 developed fever and was positive for the influenza B virus (InfB). He was isolated. Oseltamivir prophylaxis was initiated for the other athletes. On February 16, athlete 5 reported nasal congestion and was positive for RSVb. He stayed isolated in his room and his roommate, athlete 6, moved. Five days later he developed nasal congestion and was positive for RSVb. Two athletes, stayed healthy (H). (JPG 743 kb) [file 40279_2022_1660_MOESM2_ESM.jpg]
